# A Novel Long Noncoding RNA lincRNA00892 Activates CD4^+^ T Cells in Systemic Lupus Erythematosus by Regulating CD40L

**DOI:** 10.3389/fphar.2021.733902

**Published:** 2021-10-11

**Authors:** Xiao Liu, Jinran Lin, Hao Wu, Yilun Wang, Lin Xie, Jinfeng Wu, Haihong Qin, Jinhua Xu

**Affiliations:** Department of Dermatology, Huashan Hospital, Fudan University, Shanghai, China

**Keywords:** systemic lupus erythematosus, CD4^+^ T cells, B cells, long noncoding RNA, CD40L, heterogeneous nuclear ribonucleoprotein K

## Abstract

**Objective:** The mechanism of CD4^+^ T-cell dysfunction in systemic lupus erythematosus (SLE) has not been fully understood. Increasing evidence show that long noncoding RNAs (lncRNAs) can regulate immune responses and take part in some autoimmune diseases, while little is known about the lncRNA expression and function in CD4^+^ T of SLE. Here, we aimed to detect the expression profile of lncRNAs in lupus CD4^+^ T cells and explore the mechanism that how lincRNA00892 in CD4^+^ T cells is involved in the pathogenesis of SLE.

**Methods:** The expression profiles of lncRNAs and mRNAs in CD4^+^ T cells from SLE patients and healthy controls were detected by microarray. LincRNA00892 and CD40L were chosen for validation by quantitative real-time PCR (qRT-PCR). Coexpression network was conducted to predict the potential target genes of lincRNA00892. Then lincRNA00892 was overexpressed in normal CD4^+^ T cells via lentivirus transfection. The expression of lincRNA00892 was detected by qRT-PCR. The expression of CD40L was detected by qRT-PCR, western blotting, and flow cytometry, respectively. The expression of CD69 and CD23 was measured by flow cytometry. The secretion of IgG was determined by enzyme-linked immunosorbent assay (ELISA). The proteins targeted by lincRNA00892 were measured by RNA pulldown and subsequent mass spectrometry (MS). The interaction between heterogeneous nuclear ribonucleoprotein K (hnRNP K) and lincRNA00892 or CD40L was detected by RNA immunoprecipitation (RIP) assay.

**Results:** A total of 1887 lncRNAs and 3375 mRNAs were found to be aberrantly expressed in CD4^+^ T cells of SLE patients compared to healthy controls. LincRNA00892 and CD40L were confirmed to be upregulated in CD4^+^ T cells of SLE patients by qRT-PCR. The lncRNA–mRNA coexpression network analysis indicated that CD40L was a potential target of lincRNA00892. Overexpression of lincRNA00892 enhanced CD40L protein levels while exerting little influence on CD40L mRNA levels in CD4^+^ T cells. In addition, lincRNA00892 could induce the activation of CD4^+^ T cells. Furthermore, lincRNA00892 led to the activation of B cells and subsequent secretion of IgG in a CD4^+^ T-cell–dependent manner. Finally, hnRNP K was found to be among the proteins pulled down by lincRNA00892, and hnRNP K could bind to lincRNA00892 or CD40L directly.

**Conclusion:** Our results showed that the lncRNA expression profile was altered in CD4^+^ T cells of SLE. LincRNA00892 possibly contributed to the pathogenesis of SLE by targeting hnRNP K and subsequently upregulating CD40L expression to activate CD4^+^ T and B cells. These provided us a potential target for further mechanistic studies of SLE pathogenesis.

## Introduction

Systemic lupus erythematosus (SLE) is a systemic autoimmune disease, characterized by the production of autoantibodies against a wide range of self-antigens, resulting in inflammation and organ damage (Rahman and Isenberg, 2008). Although the etiology of SLE remains to be elucidated, accumulating studies have indicated that dysfunction of CD4^+^ T cells is crucial in the onset and development of SLE by facilitating lymphocytic organ infiltration and promoting B cells in producing autoantibodies that eventually lead to tissue injury (Enghard et al., 2009; Engler et al., 2011; Weinstein et al., 2012).

CD40L (also known as CD154), a member of the tumor necrosis factor superfamily, is a co-stimulator primarily expressed on activated CD4^+^ T cells (Lederman et al., 1992). It interacts with CD40, which is expressed on antigen-presenting cells (APCs), such as B cells, to provide the co-stimulatory signal of T-cell activation, thus facilitating the activation of T cells. In addition, the interaction between CD40L and CD40 can promote CD4^+^ T-cell–dependent B-cell maturation, activation, and function (Lederman et al., 1994; Cleary et al., 1995). Multiple research studies have revealed that the dysregulation of CD40L was associated with many diseases, including inflammatory responses, autoimmune diseases, and immune deficiency diseases (Elgueta et al., 2009; Liao et al., 2012). As a characteristic autoimmune disease, the pathogenesis of SLE is associated with the dysregulation of CD40L as well, since CD40L was reported to be overexpressed on T cells from both lupus-prone mice and SLE patients. In addition, CD40L-transfected normal T cells were found to induce B-cell activation, plasma cell differentiation, and subsequent IgG production, and such induction can be reversed by anti-CD40L antibody (Lettesjo et al., 2000; Lu et al., 2007; Zhou et al., 2009). Moreover, the CD40L−/− New Zealand black (NZB) mice showed a significantly decreased level of IgG autoantibodies and attenuated kidney injury (Pau et al., 2011). Therefore, CD40L serves as a potential target for SLE therapy. Dapirolizumab, a newly developed anti-CD40L antibody, showed a trend to ameliorate disease activity, such as hematuria, proteinuria, complement and dsDNA antibody levels. It is being evaluated in phase II clinical trials for SLE treatment (Narain and Furie, 2016; Touma and Gladman, 2017).

Long noncoding RNAs (lncRNAs) are a new mechanism of epigenetic regulation, which has attracted great interest in recent years. LncRNAs are more than 200 nucleotides in length and are involved in diverse biological processes. Dysregulation of lncRNAs was found to have relevance to many human diseases ranging from neurological disorders to various cancers (Faghihi et al., 2008; Gupta et al., 2010; Johnson, 2012; Pan et al., 2016). LncRNAs were also found to play important roles in regulating immune responses, including immune cell development, such as T lymphocytes (Sigdel et al., 2015). Emerging evidence suggested that lncRNA dysregulation might play a key role in autoimmune diseases such as SLE. Growth arrest–specific transcript, also known as Gas5, was found to link with increased susceptibility to SLE in mouse models (Haywood et al., 2006). LincRNA0949 and LincRNA0597 were identified to be significantly decreased in peripheral blood mononuclear cells (PBMCs) from SLE patients compared to those from rheumatoid arthritis patients and healthy controls (Wu et al., 2015). In addition, lncRNA NEAT1 was shown to be involved in the TLR4-mediated inflammatory process and contribute to the production of a number of cytokines and chemokines by regulating the MAPK signaling pathway in SLE patients (Zhang et al., 2016). However, little is known about the expression and function of lncRNAs in CD4^+^ T of SLE. LincRNA00892, a long intergenic noncoding RNA that locates in Xq26.3, contains 2886 nucleotides and 3 exons. It has not been reported to be associated with any diseases yet.

In our present study, we aimed to identify differentially expressed lncRNAs and mRNAs between CD4^+^ T cells of SLE patients and healthy controls by lncRNA and mRNA coexpression microarray. In addition, we aimed to detect how lincRNA00892 contributes to the pathogenesis of SLE by regulating the expression of CD40L in CD4^+^ T cells.

## Materials and Methods

### Subjects

In the lncRNA and mRNA coexpression microarray, peripheral blood samples were from 6 female patients (mean age 32 ± 9.8 years, range from 24 to 45 years) diagnosed with SLE according to the classification criteria of the American College of Rheumatology (Hochberg, 1997), and 6 female age-matched healthy controls. Disease activity of SLE patients was determined by the systemic lupus erythematosus disease activity index (SLEDAI) score, and the median score was 15 (range from 9 to 19). In the subsequent validation experiment, peripheral blood samples were taken from 36 SLE patients (32 females and 4 males, mean age 30 ± 12.6 years, range from 13 to 66 years), with a median SLEDAI score of 10.7 (range from 2 to 22) and 28 age- and sex-matched healthy controls. In the mechanism study, peripheral blood samples were taken from healthy controls, which were different from microarray and qRT-PCR validation experiments. This study was approved by the Independent Ethics Committee of Huashan Hospital, and written informed consents were obtained from all subjects ((2014) ethical review (No.025)).

### Separation of CD4^+^ T Cells and B Cells

PBMCs were isolated from peripheral blood samples by Ficoll-Hypaque (Sigma Aldrich, United States) density gradient centrifugation (Eppendorf, Germany). CD4^+^ T or B cells were isolated by positive selection using CD4 or CD19 magnetic beads (Miltenyi Biotec, Germany) according to the manufacturer’s instructions. Purity was evaluated by flow cytometry (purity≥ 90%, data not shown; Life technology, United States).

### RNA Extraction and Purification

Total cellular RNA was extracted using TRIzol reagent (Life technologies, United States) following the manufacturer’s instructions. The integration was checked by an Agilent Bioanalyzer 2100 (Agilent technologies, United States). Qualified total RNA was further purified by an RNeasy micro kit (QIAGEN, Germany) and RNase-Free DNase Set (QIAGEN) according to the manufacturer’s instructions.

### LncRNA and mRNA Microarray

The human 4x180k long noncoding RNA array (Agilent technologies) that included 63431 lncRNA and 39887 mRNA probes was used to determine the expression profiles of both lncRNAs and mRNAs in CD4^+^ T cells of both healthy controls and SLE patients. Each array represented all long transcripts, both protein coding mRNAs and lncRNAs in the human genome. LncRNAs were collected from the authoritative data sources including NCBI RefSeq, UCSC, RNAdb, lncRNAs from the literature, and UCRs. Each transcript was represented by 1–5 probes to improve statistical confidence.

### RNA Labeling and Array Hybridization

Microarray hybridization was performed by Shanghai Biotechnology Corporation (Shanghai, China). In brief, total RNA was amplified and labeled by a Low Input Quick Amp Labeling Kit with one color (Agilent technologies) following the manufacturer’s instructions. The labeled cRNAs were hybridized onto the human LncRNA array slides in a hybridization oven. After hybridization, the slides were washed in stain dishes (Thermo Shandon, United States). Then the arrays were scanned by the Agilent Scanner (Agilent technologies) with default settings. Data were extracted with Agilent Feature Extraction Software v10.7.3.1, and quantile normalization and subsequent data processing were carried out using the GeneSpring GX v11.5.1 software package (Agilent Technologies). Differentially expressed lncRNAs and mRNAs were identified through filtering with the threshold setting of fold change ≥2.0 or ≤0.5 and *p* value <0.5. Hierarchically clustering analysis was conducted to show the differently expressed lncRNAs or mRNAs and the relationships between these transcripts. The expression data of both lncRNAs and mRNAs have been uploaded onto the Gene Expression Omnibus (GEO), with the accession number GSE181500.

### Analysis of lncRNA–mRNA Regulatory Network

To show that the lncRNAs directly regulated the expression of targeted mRNAs, we superimposed lncRNA target predictions onto the lncRNA–mRNA correlation network. Pearson correlation analysis was conducted to estimate the significance of the correlation between each pair of lncRNA and mRNA. The paired lncRNA and mRNA was included in the network when the Pearson correlation coefficient between them was more than 0.95, and the *p* value was less than 0.05. The resulting network was defined as an lncRNA–mRNA regulatory network and visually presented with Cytoscape v3.1.0 software. A direct connection was placed from an lncRNA to an mRNA using the solid line.

### Quantitative Real-Time PCR Validation

Total RNA was extracted from CD4^+^ T cells of SLE patients (*n* = 36) and healthy controls (*n* = 28) using TRIzol reagent, as indicated before, and subsequently reverse-transcribed into complementary DNA (cDNA) via a PrimeScript^®^ RT reagent kit (Takara, Japan) on an S1000™ Thermal Cycler (BioRad, United States). Then the qRT-PCR was performed via an SYBR^®^ Premix Ex TaqTM (Takara) on a QuantStudio™ 6 Flex Real-Time PCR System (Life technology). The used primers were listed in Supplementary Table S2. All primers were purchased from BioTNT (China). The relative fold change was calculated using the 2^−ΔΔCt^ method normalized to β-actin.

### Lentivirus Preparation

The plasmid FUGW-GRK5-IRES-EGFP was used as the vector to construct the lentivirus vector containing lincRNA00892. The human genomic lincRNA00892 fragment, which was synthesized *in vitro*, was digested with XbaI and BamHI (NEB, United States) and then was ligated with T4 ligase (Takara). The reconstructed plasmid containing lincRNA00892 was verified with Sanger sequencing. After that, 293T cells were cotransfected with the control vector or reconstructed vector containing lincRNA00892 fragment. After 24 h or 48 h, the collected culture medium was filtered through the 0.45-um filter and subsequently incubated with PEG8000 overnight, followed by centrifugation at 4000 g at 4°C for 20 min. Finally, the lentiviruses were titrated by a Quick Titer™ Lentivirus Titer Kit (Cell Biolabs, United States) according to the manufacturer’s instruction.

### Cell Culture and Lentivirus Transfection

The Jurkat cells were purchased from ATCC and cultured in RPMI 1640 medium supplemented with 10% fetal bovine serum (FBS) and 1% penicillin/streptomycin (all from Life Technologies) at 37°C in a 5% CO_2_ humidified incubator. For lentivirus transfection, the Jurkat cells were incubated with 5ug/ml polybrene (Sigma Aldrich) and previously prepared lentiviruses with control vector or reconstructed vector containing lincRNA00892 fragment (*MOI* = 50) in 1 ml of RPMI 1640 medium without FBS and penicillin/streptomycin at 37°C in a 5% CO_2_ humidified incubator for 12–24 h; then the culture medium with lentiviruses and polybrene was replaced by RPMI 1640 medium with 10% FBS and 1% penicillin/streptomycin. Another 48–60 h later, the Jurkat cells transfected with lentiviruses were harvested for western blotting, qRT-PCR, and flow cytometry.

The CD4^+^ T cells isolated from healthy controls with a purity of over 90% were cultured in 6-well plates (1×10^6^/well) or 96-well plates (1×10^4^/well) in OpTmizer™ CTS™ T-Cell Expansion SFM supplemented with 1% penicillin/streptomycin and 1× GlutaMAX™ Supplement (all from Life Technologies) at 37°C in a 5% CO_2_ humidified incubator. For lentivirus transfection, the isolated CD4^+^ T cells were incubated with 5 ug/ml polybrene and previously prepared lentiviruses with control vector or reconstructed vector containing lincRNA00892 fragment (*MOI* = 50) in 1 ml of OpTmizer™ CTS™ T-Cell Expansion SFM supplemented with 1× GlutaMAX™ Supplement at 37°C in a 5% CO_2_ humidified incubator for 12–24 h; then the culture medium with lentiviruses and polybrene was replaced by fresh medium (OpTmizer™ CTS™ T-Cell Expansion SFM supplemented with 1% penicillin/streptomycin and 1× GlutaMAX™ Supplement). Another 48–60 h later, the CD4^+^ T cells transfected with lentiviruses were harvested for western blotting, qRT-PCR, flow cytometry, and coculturing with B cells.

### T-Cell and B-Cell Coculture

The B cells isolated from healthy controls with a purity of over 90% were cocultured with lentivirus-transfected CD4^+^ T cells in OpTmizer™ CTS™ T-Cell Expansion SFM supplemented with 1% penicillin/streptomycin and 1× GlutaMAX™ Supplement in both 6-well and 96-well plates at a ratio of 1:4 or 1:1 for 3 days. Finally, the cells in the 6-well plate were harvested for flow cytometry, and the supernatants in the 96-well plate were harvested for ELISA.

### Western Blotting

The Jurkat and CD4^+^ T cells, transfected with lentiviruses, were lysed into RIPA lysis buffer (Beyotime biotechnology, China) and then centrifuged at 4°C at a speed of 14000 rpm for 15 min. Total amounts of 30 μg of cellular proteins were separated via electrophoresing on 10% SDS-PAGE (Beyotime biotechnology) and transferred to the polyvinylidene difluoride (PVDF) membranes (Millipore, United States). After that, the PVDF membranes were blocked with 5% nonfat milk for 1 h at room temperature, followed by an incubating step with primary antibody against CD40L (Abcam, United States) or β-actin (Abcam) overnight at 4°C. On the second day, the membranes were incubated with secondary antibody from rabbits or mice conjugated with horseradish peroxidase (HRP) at room temperature for 1 h. In the end, the bands were detected by ECL technology (Fujifilm LAS-3000, Japan). The band intensities were quantified by Quantity One Software (BioRad). β-actin was regarded as reference, and the relative expression levels were therefore calculated.

### Flow Cytometry

The lentivirus transfected Jurkat cells and CD4^+^ T cells or cocultured CD4^+^ T and B cells were harvested by centrifuging at 4°C, 300 g for 10 min and subsequently washing with phosphate-buffered saline (PBS) PH7.4. Then the lentivirus-transfected Jurkat cells and CD4^+^ T cells were incubated with phycoerythrin (PE)-conjugated anti-human CD40L (BD Pharmingen, United States) and allophycocyanin (APC)-conjugated anti-human CD69 (BD Pharmingen) in staining buffer (PBS supplemented with 1% bovine serum albumin (BSA)) for 30 min at 4°C in the dark. The cocultured CD4^+^ T and B cells were incubated with APC-conjugated anti-human CD23 (BD Pharmingen) and PerCP-Cy5.5-conjugated anti-human CD19 (BD Pharmingen) for 30 min at 4°C in the dark. Next, the labeled cells were washed three times with staining buffer and resuspended in staining buffer at 1×10^5^/200 ul. Finally, the data were obtained by a FACS system (Life technology). The data were analyzed by FlowJo software version 6.0 (Tree Star, Inc.).

### Enzyme-Linked Immunosorbent Assay

The IgG levels in the supernatants of CD4^+^ T- and B-cell coculture system were measured using a RayBio Human IgG ELISA Kit (RayBiotech, United States), according to the instructions of the manufacturer. The antibody specific for human IgG was coated onto the 96-well plate overnight; then 100 ul of standard or sample diluted 5 times was directly added to the corresponding wells, followed by 2.5-h incubation at room temperature with gentle shaking. After 4-time washing with 1× wash solution, 100 ul of prepared biotinylated antibody was added to each well and incubated at room temperature for 1 h with gentle shaking, followed by 4-time washing. In the next step, 100 ul of the prepared streptavidin solution was added and incubated at room temperature for 45 min with gentle shaking, followed by 4-time washing. After that, 100 ul of TMB One-Step Substrate Reagent was added to each well and incubated at room temperature in the dark with gentle shaking for 30 min. Finally, 50 ul of stop solution was added to each well. The absorbance was read at 450 nm immediately by the Infinite F200 Pro microplate reader (TECAN, Switzerland).

### RNA Pulldown and Mass Spectrometry

The sense and anti-sense lincRNA00892–containing T7 promoters were developed using pGEM-T Easy Vector Systems (Promega, United States) following the manufacturer’s instruction. The primers used were listed in Supplementary Table S2. The lincRNA00892 was transcribed *in vitro* with the help of T7 RNA polymerase (Roche, Switzerland) and labeled with biotin using Biotin RNA labeling Mix (Roche) according to the manufacturer’s instruction. Then the biotin-labeled lincRNA00892 was incubated with streptavidin magnetic beads at 4°C overnight. The biotinylated lincRNA00892–streptavidin magnetic beads mixture was incubated with the cell lysates containing about 1 mg of protein at room temperature for 1 h to capture the proteins. After 3-time washing, the RNA–bead–protein mixture was electrophoresed on SDS-PAGE and stained in Janesen. The gels showed significant differences in silver staining were took out and subjected to MS.

### RNA-Binding Protein Immunoprecipitation

RIP assay was conducted to determine the interaction between heterogeneous nuclear ribonucleoprotein K (hnRNP K) and lincRNA00892 or CD40L using an EZ-Magna RIP Kit (Millipore) following the manufacturer’s instruction. In brief, 1×10^7^ CD4^+^ T cells isolated from healthy controls were lysed in RIPA buffer overnight and centrifuged at 14000 rpm at 4°C for 10 min to collect the supernatants. The beads were incubated with 5 μg of anti-hnRNP K, anti-SNRNP70 (positive control), or IgG (negative control) antibodies at room temperature for 30 min. Then the mixtures were washed with RIP wash buffer and resuspended in 860 μL of RIP wash buffer, 35 μL of 0.5 M EDTA, 5 μL of RNase inhibitor, and 100 μL of protein supernatants. Next, the protein–bead–antibody mixtures were incubated at 4°C overnight followed by 6-time washing with RIP wash buffer. The RNA was eluted from the protein–bead–antibody mixtures and reverse-transferred into cDNA. Finally, the coprecipitated lincRNA00892 or CD40L from the protein–bead–antibody mixtures was measured by qRT-PCR. The primers used were listed in Supplementary Table S2.

### Statistical Analysis

All the results were expressed as mean ± standard deviation. Statistical analysis was done with Student’s t-test for comparison of two groups, and analysis of variance for multiple comparisons. Differences with *p* < 0.05 were considered statistically significant. The statistical significance of microarray result was analyzed by fold change and Student’s t-test. The threshold value we used to screen differentially expressed lncRNAs and mRNAs is set as a fold change ≥2.0 or ≤0.5 (*p* < 0.05).

## Results

### Differentially Expressed lncRNAs and mRNAs in CD4^+^ T Cells of SLE Patients

To profile differentially expressed lncRNAs, we performed a genome-wide analysis of lncRNA and mRNA expressions in CD4^+^ T cells from 6 SLE patients and 6 healthy controls and found that 1887 lncRNAs were differentially expressed between SLE patients and healthy controls (Supplementary Figure S1A). Among them, 1083 lncRNAs were upregulated, and 804 lncRNAs were downregulated in CD4^+^ T cells from SLE patients as compared to those from healthy controls (Supplementary Figure S1B).

Using the same data as before, we identified 3375 mRNAs to be significantly and differentially expressed in CD4^+^ T cells from SLE patients as compared to healthy controls (Supplementary Figure S1C), of which 1438 mRNAs were upregulated and 1937 mRNAs were downregulated (Supplementary Figure S1D).

**FIGURE 1:**
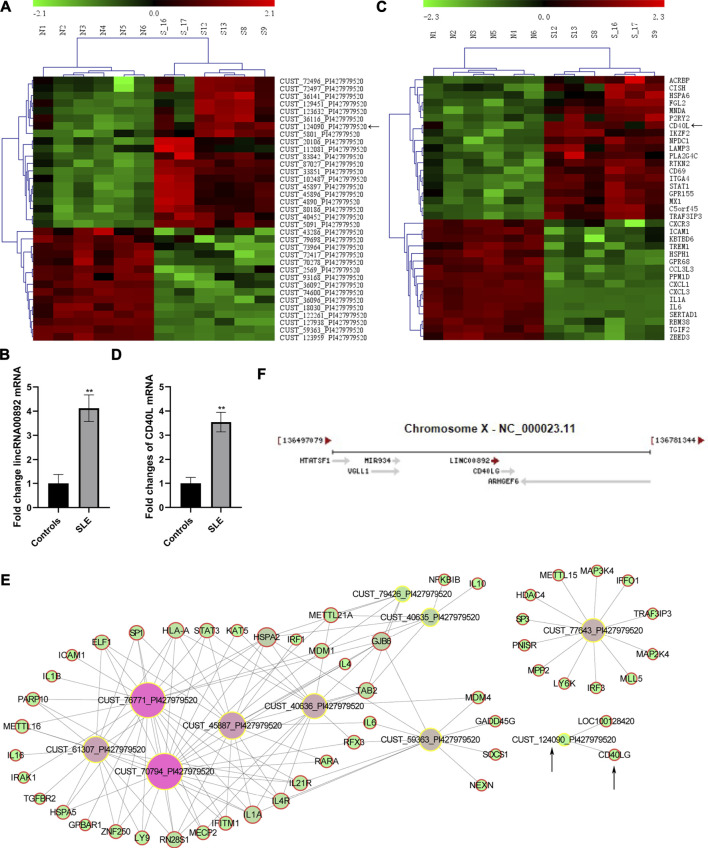
LncRNA CUST124090 (lincRNA00892) and CD40L were upregulated in CD4^+^ T cells of SLE patients. **(A)** Hierarchically clustered heatmaps of some lncRNAs that were upregulated in CD4^+^ T cells in SLE patients (*n* = 6) as compared to healthy controls (*n* = 6). **(B)** qRT-PCR was used to verify the expression of lincRNA00892 in CD4^+^ T cells of SLE patients (*n* = 36) and healthy controls (*n* = 28). **(C)** Hierarchically clustered heatmaps of some mRNAs that were upregulated in CD4^+^ T cells of SLE patients (*n* = 6) as compared to healthy controls (*n* = 6). **(D)** qRT-PCR was performed to detect the expression of CD40L in CD4^+^ T cells of SLE patients (*n* = 36) and healthy controls (*n* = 28). **(E)** Coexpression network analysis of some lncRNAs and predicted targeted mRNAs. **(F)** The location of both lincRNA00892 and CD40L in chromosome X. **p < 0.01, *p < 0.05. The results are expressed as mean ± standard deviation. Student’s t-test was used for comparison of two groups.

LincRNA00892 and CD40L were both upregulated in CD4^+^ T cells from SLE patients. Among the 1887 lncRNAs identified by our genome-wide analysis, we chose lncRNA CUST124090 which was then proved to be lincRNA00892 to further investigate the molecular mechanisms that how it contributes to the pathogenesis of SLE. As revealed in the genome-wide analysis and subsequent qRT-PCR validation, we found that the expression of lincRNA00892 was much higher in SLE patients (*n* = 36) than healthy controls (*n* = 28) (*p* < 0.05, Figures 1A,B). Coexpression network analysis indicated that CD40L was a potential target of lincRNA00892 (Figure 1E). Moreover, lincRNA00892 was shown to be closely located with CD40L in chromosome X (Figure 1F). In addition, the expression levels of CD40L were proved to be upregulated in CD4^+^ T cells of SLE patients by our microarray and qRT-PCR validation in samples from 36 SLE patients and 28 healthy controls as well (*p* < 0.05, Figures 1C,D). Therefore, we hypothesized that lincRNA00892 contributed to the pathogenesis of SLE via mediating the expression of CD40L and subsequently activating CD4^+^ T and B cells.

### LincRNA00892 Promoted the Expression of CD40L in Both Jurkat and Primary CD4^+^ T Cells

**FIGURE 2:**
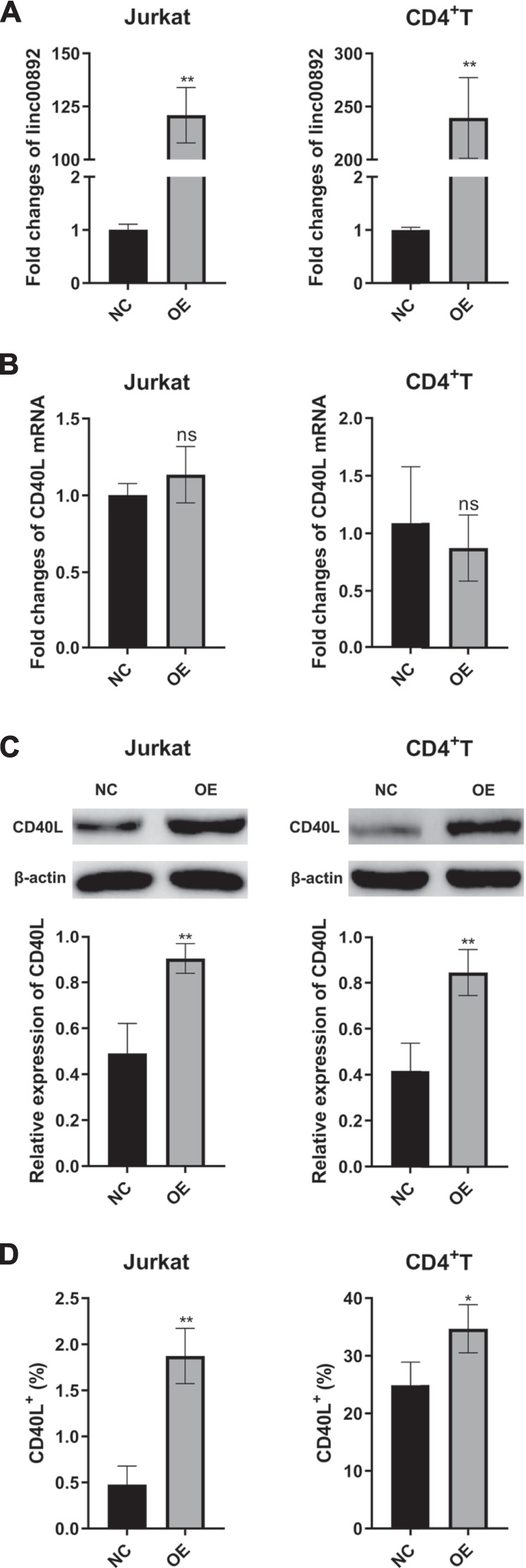
LincRNA00892 enhanced CD40L expression in Jurkat and primary CD4^+^ T cells. **(A)**, **(B)**: The expression of lincRNA00892 **(A)** and CD40L **(B)** in Jurkat and primary CD4^+^ T cells transfected with lentiviruses with control vector or vector containing the lincRNA00892 fragment was defined by qRT-PCR. **(C)** The protein levels of CD40L in the negative control and lincRNA00892 overexpression group in both Jurkat and primary CD4^+^ T cells were confirmed by western blotting. **(D)** The level of CD40L expressed on the surface of both Jurkat and primary CD4^+^ T cells in both negative control and lincRNA00892 overexpression group were performed by flow cytometry. NC = negative control, OE = overexpression, NS = no statistical difference. **p < 0.01, *p < 0.05. The results are expressed as mean ± standard deviation. Student’s t-test was used for comparison of two groups.

As shown in Figure 2A, lentiviruses with control vector or reconstructed vector containing lincRNA00892 fragment were successfully transfected into both Jurkat and primary normal CD4^+^ T cells, since the expression of lincRNA00892 in both Jurkat and primary CD4^+^ T cells transfected with lentivirus with lincRNA00892 fragment was proved to be much higher than expression of those transfected with control vectors by qRT-PCR (*p* < 0.05, Figure 2A). Then we found significantly increased protein levels of CD40L when lincRNA00892 was overexpressed in both Jurkat and primary CD4^+^ T cells (*p* < 0.05, Figures 2C,D), while no significant difference was found in the mRNA levels of CD40L between control and lincRNA00892 overexpression groups (*p* > 0.05, Figure 2B). These results indicated that lincRNA00892 might mediate CD40L expression in a posttranscriptional way.

### LincRNA00892 Activated CD4^+^ T Cells and Subsequently Activated Primary B Cells in a CD4^+^ T-Cell–Dependent Manner

**FIGURE 3:**
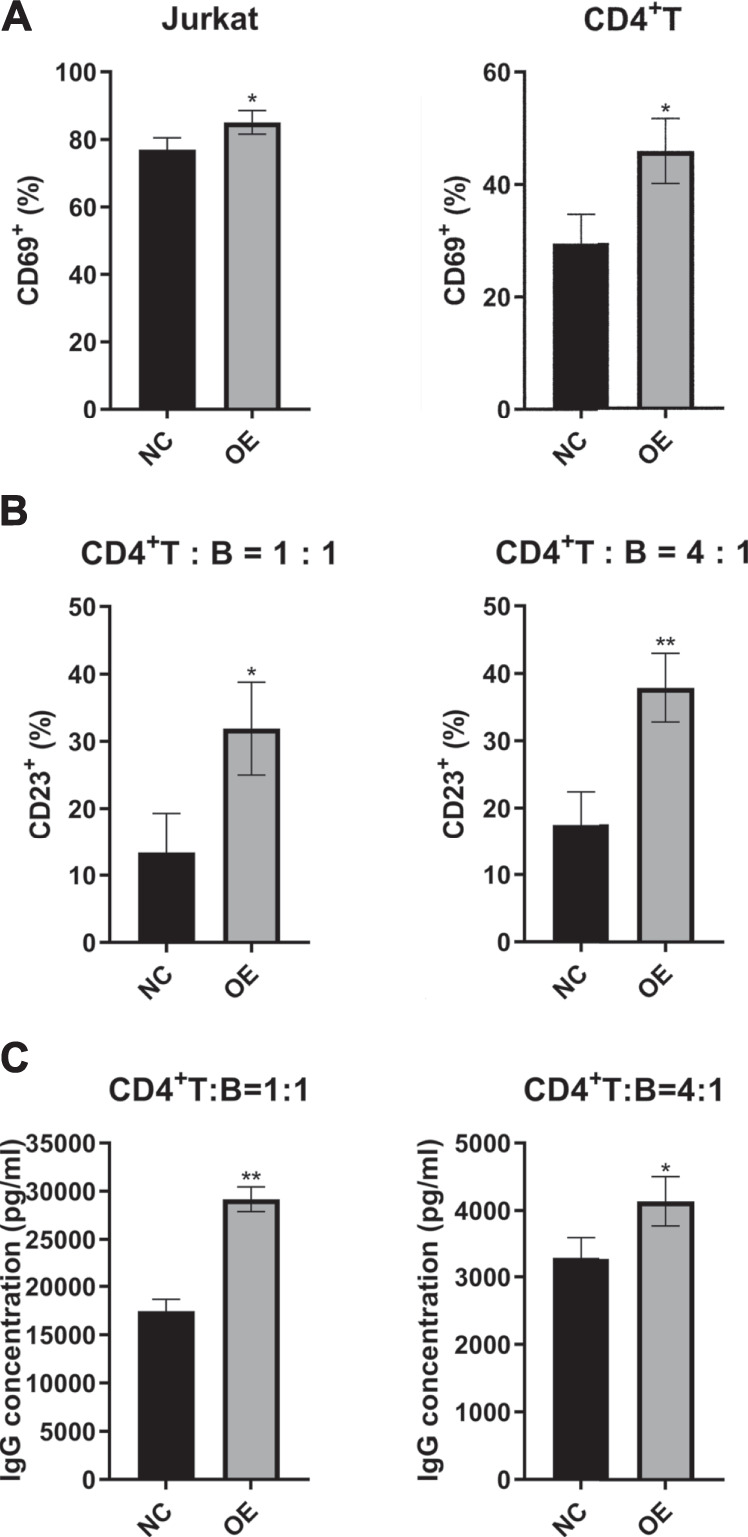
LincRNA00892 promoted the activation of CD4^+^ T cells and subsequent activation of B cells. **(A)** The CD69 levels expressed on the surface of both Jurkat and primary CD4^+^ T cells in negative control and lincRNA00892 overexpression groups were measured by flow cytometry. **(B)** The CD23 levels expressed on the surface of B cells cocultured with primary CD4^+^ T cells transfected with control vector or vector containing lincRNA00892 fragment were confirmed by flow cytometry. **(C)** The primary CD4^+^ T cells that transfected with control vector or vector that containing lincRNA00892 fragment were cocultured with B cells at a ratio of 1:1 or 4:1 for 3 days. The levels of IgG secreted by B cells were determined by ELISA. NC = negative control, OE = overexpression. **p < 0.01, *p < 0.05. The results are expressed as mean ± standard deviation. Student’s t-test was used for comparison of two groups.

As we know, CD40L is a co-stimulator mainly expressed on active T cells and promotes T-cell activation and T-cell–dependent B-cell maturation, activation, and function. Since overexpression of lincRNA00892 in normal CD4^+^ T cells was revealed before to increase the expression of CD40L, we decided to determine whether overexpression of lincRNA00892 could activate T cells. We examined the expression levels of CD69 (a marker of T-cell activation) on the surface of both Jurkat and primary normal CD4^+^ T cells by flow cytometry and found that CD69 levels were much higher in the lincRNA00892 overexpression group than in the control group (*p* < 0.05, Figure 3A). Next, we explored whether lincRNA00892 could promote the activation and secretion of IgG by B cells in a CD4^+^ T–B-cell coculture system. We found that the expression of CD23 (a marker of B cell activation) on the surface of B cells was much higher in normal B cells, which were cocultured with CD4^+^ T cells transfected with lentivirus with lincRNA00892 fragment at a ratio of 1:1 or 1:4 for 3 days (*p* < 0.05, Figure 3B). Furthermore, the IgG levels secreted by B cells cocultured with lincRNA00892-overexpressed CD4^+^ T cells were much higher than those from B cells cocultured with control vector–transfected CD4^+^ T cells (*p* < 0.05, Figure 3C). Thus, we concluded that overexpression of lincRNA00892 was able to activate CD4^+^ T cells and subsequently promote the activation and IgG secretion of B cells in a CD4^+^ T-cell–dependent manner.

### LincRNA00892 Mediated CD40L Expression by Directly Binding to Heterogeneous Nuclear Ribonucleoprotein K in CD4^+^ T Cells

**FIGURE 4:**
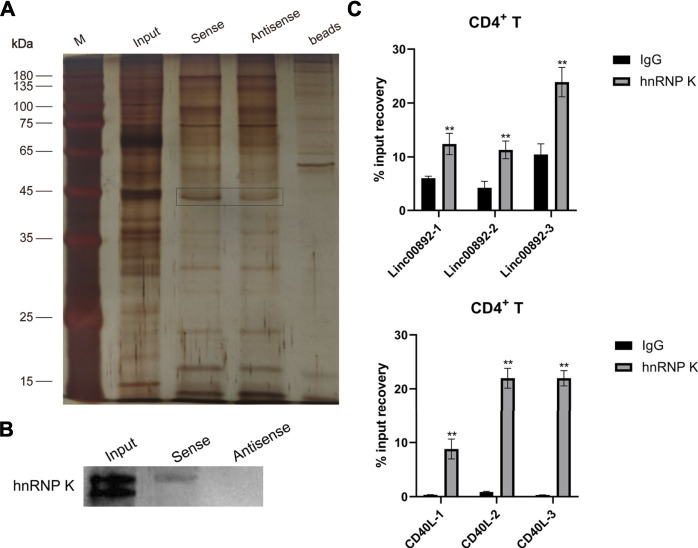
LincRNA00892 mediates CD40L expression by targeting hnRNP K in primary CD4^+^ T cells. **(A)** RNA pulldown assay was used to identify the proteins interacted with lincRNA00892. Silver staining of the proteins pulled down by lincRNA00892. **(B)** The expression of hnRNP K in the gel pulled down by control, sense, and anti-sense lincRNA00892 was confirmed by western blotting. **(C)** The interaction between hnRNP K and lincRNA00892 (up) and CD40L (down) was identified by RIP. IgG was used as the negative control. **p < 0.01, *p < 0.05. The results are expressed as mean ± standard deviation. Student’s t-test was used for comparison of two groups.

Given that lncRNAs could function by recruiting and interacting with RNA-binding proteins (Zhang et al., 2017), we tried to identify lincRNA00892-interacting proteins by the RNA pulldown assay and subsequent MS. As shown in Figure 4A, distinct bands were found between sense and anti-sense lincRNA00892. The differently expressed proteins were identified by MS (Supplementary Table S1). HnRNP K is an RNA-binding protein (RBP) that plays an important role in posttranscriptional regulation which lncRNAs are usually involved in (Pereira et al., 2017). We hypothesized that lincRNA00892 regulated CD40L expression by recruiting and binding to hnRNP K. We confirmed the distinct hnRNP K expression in sense and anti-sense lincRNA00892 by western blotting (Figure 4B). In order to further verify our hypothesis, RIP was conducted to confirm the interaction between hnRNP K and lincRNA00892 or CD40L. As shown in Figure 4C, both lincRNA00892 and CD40L mRNA were enriched by hnRNP K antibody as compared to negative control (IgG) in primary normal CD4^+^ T cells, indicating that lincRNA00892 recruited hnRNP K to regulate CD40L expression through a posttranscription way.

## Discussion

Increasing evidence has pointed to the critical regulatory roles of lncRNAs in immune cellular biological processes including T lymphocyte differentiation and activation in recent years. Genome-wide expression analyses revealed the presence of hundreds of lncRNAs in CD8^+^ T cells from human and mouse spleens (Pang et al., 2009). T-cell–expressed lncRNA–Tmevpg1 has antiviral activity by promoting the release of IFN-γ (Mourtada-Maarabouni et al., 2008). LncRNA GAS5 can regulate T-cell growth (Jones and Flavell, 2005). After activation, CD4^+^ T cells can express lncRNA-NTT (Vigneau et al., 2003) or lncRNA-BI (Liu et al., 1997). It has also been demonstrated that Lnc-DC, a specific regulator of dendritic cell (DC) differentiation and function, may have a potential role in clinical diseases involving DC dysfunction and may have an influence on the activation of the CD4^+^ T-cell response (Shirasawa et al., 2004). In addition to the biological regulation of lncRNAs on immune systems, several studies have also shown that abnormal expressions of lncRNAs may play a pivotal role in some autoimmune diseases, including SLE (Haywood et al., 2006; Wu et al., 2015; Zhang et al., 2016).

In our present work, we first explored the lncRNA and mRNA expression profiles in CD4^+^ T cells of SLE patients and healthy controls using microarray technology. From these microarray data, we found that 1887 lncRNAs and 3375 mRNAs were differentially expressed in lupus CD4^+^ T cells compared to the healthy controls. Among these lncRNAs, 1083 lncRNAs were upregulated, and 804 lncRNAs were downregulated.

LincRNA00892 is a long intergenic noncoding RNA that is located in Xq26.3. It has not been reported to be associated with any physiological or pathological processes yet. In our present study, we first found that lincRNA00892 was upregulated in SLE patients as compared to healthy controls, indicating that it might participate in the pathogenesis of SLE. CD40L is a co-stimulator expressed on active T cells which facilitates T-cell–dependent B-cell activation, maturation, and function. It has been reported to contribute to the onset and development of SLE. In accordance with these findings, our genome-wide analysis of mRNA expressions in CD4^+^ T cells and subsequent qRT-PCR validation of microarray results both revealed that CD40L was significantly upregulated in SLE patients, indicating it as a pathogenic factor in SLE. In addition, in the coexpression network analysis, CD40L was predicted to be a potential direct target of lincRNA00892. It is highly possible that lincRNA00892 contributes to the pathogenesis of SLE via upregulating CD40L and subsequently activating T cells and B cells. In accordance with this hypothesis, we revealed that overexpression of lincRNA00892 could upregulate the expression of CD40L and activate CD4^+^ T cells. In addition, lincRNA00892 overexpression activated B cells and promoted the IgG secretion in a CD4^+^ T-cell–dependent manner. Therefore, we came to the conclusion that lincRNA00892 might be involved in SLE through inducing the expression of CD40L and subsequently activating CD4^+^ T and B cells.

As indicated before, lncRNA is a newly discovered mechanism of epigenetic regulation with a length of over 200 bp and cannot code any protein (Curtale and Citarella, 2013). Some lncRNAs are reported to play an important role in gene imprinting, activation, and repression (Ponting et al., 2009; Engreitz et al., 2013; Maass et al., 2014; Yang et al., 2015). Some lncRNAs are revealed to play a pivotal role in the transcription level via binding to certain DNA or protein to regulate the localization of transcription factors and subsequent transcript elongation (Ponting et al., 2009; Wang et al., 2011; Vance and Ponting, 2014). In addition, some lncRNAs play an essential role in the posttranscriptional processes, such as alternative splicing, RNA editing, transport, degradation, and translation (Tripathi et al., 2010; Mourtada-Maarabouni and Williams, 2013; Shi et al., 2013). In our present study, we found that the protein levels of CD40L were significantly upregulated in both Jurkat and primary normal CD4^+^ T cells that overexpressed lincRNA00892, while the mRNA levels of CD40L showed no difference. Therefore, we deduced that lincRNA00892 played an important role in the posttranscriptional regulation of CD40L. To further confirm the regulation of lincRNA00892 on CD40L expression, the RNA pulldown assay and subsequent MS were conducted. However, CD40L was not among the proteins bound to lincRNA00892 directly. Therefore, other factors might be involved in the regulation of CD40L expression by lincRNA00892.

HnRNPs are a series of RBPs that bind to newly formed transcripts in the nucleus to assist the transcription, stabilization, and translation of mRNA, thus regulating gene expression (Dreyfuss et al., 1993; Geuens et al., 2016). Recently, they have been reported to interact with lncRNAs to contribute to various pathogenic disorders, such as tumorigenesis (Zhu et al., 2019). Geng et al. revealed that lncRNA PSTAR could bind to hnRNP K to enhance its SUMOylation, thus strengthening the interaction between hnRNP K and p53 to promote the accumulation and transactivation of p53 (Qin et al., 2020). LncRNA CASC11 was demonstrated to target hnRNP K to activate WNT/β-catenin signaling in colorectal cancer cells (Zhang et al., 2016). In accordance with these findings, we found that lincRNA00892 could bind to hnRNP K directly. CD40L was also reported to be regulated by different hnRNPs, such as hnRNP L, at the translational level (Hamilton et al., 2008; La Porta et al., 2016). As an important member of the hnRNP family, hnRNP K has multiple roles in mediating the transcription, splicing, mRNA silencing, mRNA stabilization, and translation (Habelhah et al., 2001; Stains et al., 2005; Fukuda et al., 2009; Cao et al., 2012; Fan et al., 2015). However, the role of hnRNP K in the regulation of CD40L expression remains unclear. In our present study, we revealed that hnRNP K could bind to CD40L directly. Therefore, we concluded that lincRNA00892 mediated CD40L expression by enrolling hnRNP K to bind to CD40L and subsequently mediate CD40L expression at the posttranscriptional level in primary CD4^+^ T cells. Sincerely, more research studies are needed to further figure out how hnRNP K regulates CD40L expression at the posttranscription level.

In conclusion, we identified a series of new SLE-associated lncRNAs, including lincRNA00892. Our mechanism study demonstrated that lincRNA00892 was involved in the pathogenesis of SLE via mediating the expression of CD40L and subsequent activation of CD4^+^ T and B cells. In addition, we found that lincRNA00892 mediated CD40L expression through enrolling hnRNP K to bind to CD40L, thus regulating the translation of CD40L.

## Data Availability

The datasets presented in this study can be found in online repositories. The names of the repository/repositories and accession number(s) can be found in the article/Supplementary Material.
