# Supplementary figures and images for: A Novel Long Noncoding RNA lincRNA00892 Activates CD4+ T Cells in Systemic Lupus Erythematosus by Regulating CD40L
Source: Front Pharmacol. 2021 Oct 11;12:733902. doi: 10.3389/fphar.2021.733902 (PMC8543062; doi:10.3389/fphar.2021.733902)

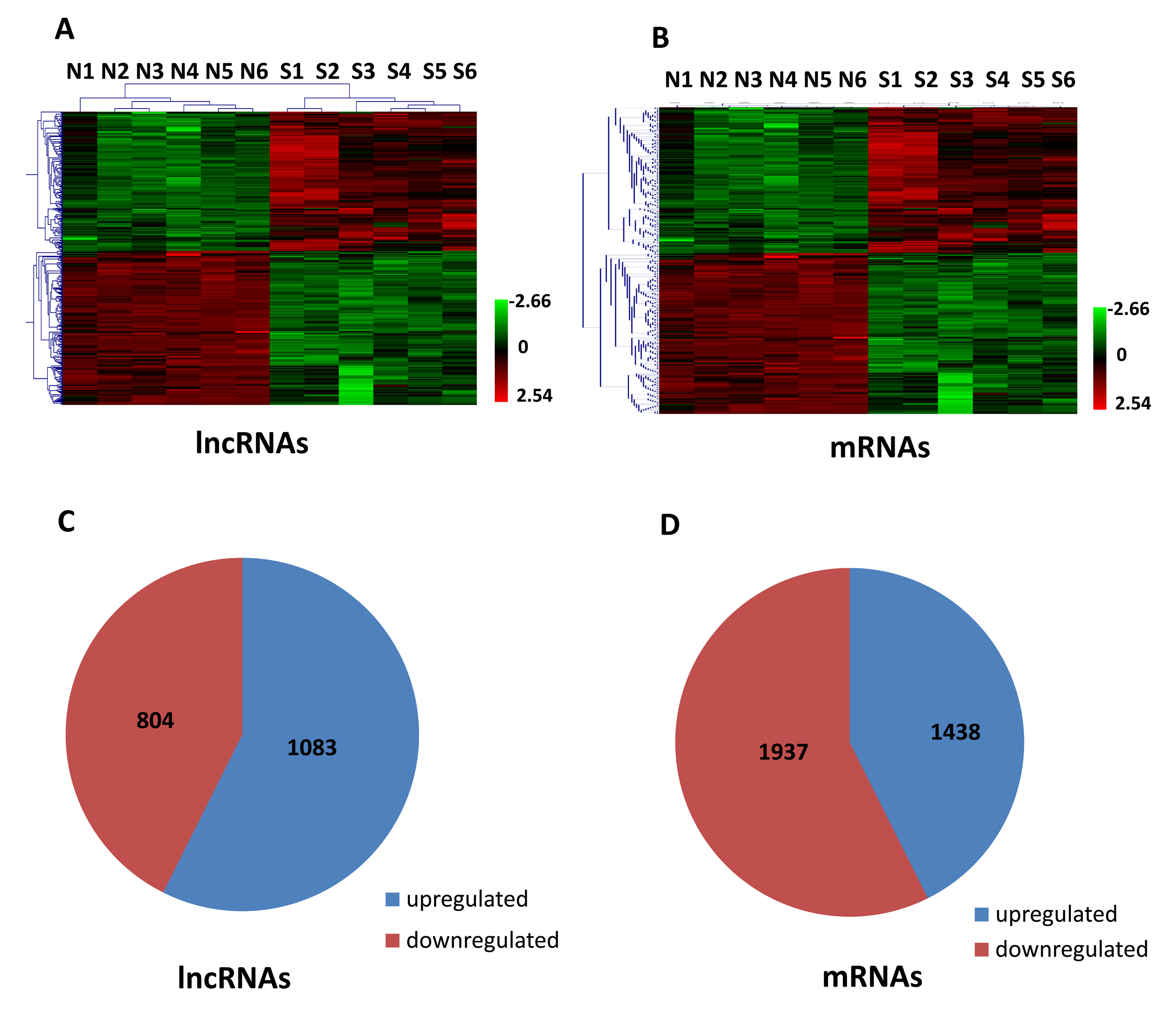

Supplement: Supplementary file 1 [file Image1.TIF]
