# Supplementary material for: A Novel Long Noncoding RNA lincRNA00892 Activates CD4+ T Cells in Systemic Lupus Erythematosus by Regulating CD40L
Source: Front Pharmacol. 2021 Oct 11;12:733902. doi: 10.3389/fphar.2021.733902 (PMC8543062; doi:10.3389/fphar.2021.733902)
Supplement: Supplementary file 2 [file Table2.docx]

Table S2 Primers used in qRT-PCR, RNA pull-down and RIP

| **qRT-PCR** | | |
| --- | --- | --- |
| Gene | Primers | Seqence |
| LincRNA00892 | F | CAGCACCTGGCTCACATTA |
|  | R | CTGGATTGGAAAGAAGCAACT |
| CD40L | F | AACGAGAAGGGTCCTTATCC |
|  | R | CCAAAGTGTTGCTCATGGTG |
| β-actin | F | GCACCACACCTTCTACAATGAGC |
|  | R | GGATAGCACAGCCTGGATAGCAAC |
| **RNA Pull-down** | | |
| lincRNA00892 | F | AGATGCAGACATGGCTGGATG |
|  | R | TGATTTATGGGTTATTGTTTATTTTCCATTA |
| lincRNA00892-sense | F | **AAAA**TAATACGACTCACTATAGGAGATGCAGACATGGCTGGATG |
|  | R | TGATTTATGGGTTATTGTTTATTTTCCATTA |
| lincRNA00892-antisense | F | AGATGCAGACATGGCTGGATG |
|  | R | **AAAA**TAATACGACTCACTATAGTGATTTATGGGTTATTGTTTATTTTCCATTA |
| **RIP** | | |
| lincRNA00892 | -1F | GATGCAGACATGGCTGGATG |
|  | -1R | catcagctcccacccactg |
|  | -2F | GCTGCATGACCATGGGC |
|  | -2R | acacttctttgaatcagttcatttgttc |
|  | -3F | AGATCCACATGAAGAATCACACATATC |
|  | -3R | ttatgggttattgtttattttccattagtg |
| CD40L | -1F | agtaaggtggccactttgacagtc |
|  | -1R | GTCCAGTGGCCGCAGATC |
|  | -2F | caatccattcacttgggagga |
|  | -2R | CTCCACCACAGCCTGCAA |
|  | -3F | tgaattctgagtaaacagcagataacttg |
|  | -3R | ACCCAGAGTCAACCATAACTGAATG |
| U1 (control) | F | GGGAGATACCATGATCACGAAGGT |
|  | R | CCACAAATTATGCAGTCGAGTTTCCC |
